# Shear Strength of Externally U-Bonded Carbon Fiber-Reinforced Polymer High-Strength Reinforced Concrete

**DOI:** 10.3390/ma14133659

**Published:** 2021-06-30

**Authors:** Basil Ibrahim, Moussa Leblouba, Salah Altoubat, Samer Barakat

**Affiliations:** Department of Civil & Environmental Engineering, College of Engineering, University of Sharjah, Sharjah P.O. Box 27272, United Arab Emirates; basil.ibrahim@usherbrooke.ca (B.I.); saltoubat@sharjah.ac.ae (S.A.); sbarakat@sharjah.ac.ae (S.B.)

**Keywords:** shear, strengthening, ductility, FRP, interaction, ACI, *fib*

## Abstract

In this paper, we investigate the contribution of Fiber-Reinforced Polymer (FRP) to the load-carrying capacity of shear-strengthened Reinforced Concrete (RC) beams. Specifically, the investigation is focused on the FRP’s contribution in the presence and absence of shear stirrups. To this end, two sets of full-scale RC beam specimens were tested to failure in a simply supported setup. Set 1 consisted of specimens without shear stirrups whereas Set 2 included steel stirrups spaced at 170 mm. One and two layers of FRP discrete strips were bonded to the beams in a U-jacketing configuration. To investigate the contribution of FRP and its interaction with the stirrups, two different locations were considered when bonding the FRP strips: between the stirrups (referred to as Off-beams) and at the same level of the stirrups (referred to as On). Results of the experimental program showed that strengthening the beams with two layers of FRP does not necessarily translate to improved capacity. Furthermore, the location of FRP strips with respect to the location of shear stirrups has an influence on the beam’s overall behavior, especially its displacement ductility. This is an important parameter to consider to avoid premature failure of RC members. Test results were then used to assess the performance and accuracy of the predictions of ACI PRC-440.2-17 and *fib*-TG9.3. Both design codes were found to be conservative with an average prediction-to-test ratio of 0.7.

## 1. Introduction

As a shear strengthening technique for reinforce concrete (RC) members, the use of externally bonded (EB) fiber-reinforced polymer (FRP) composites is majorly driven by the desirable increase in both the load capacity of the strengthened member and its deflection ductility before failure. This can be achieved by the mechanism with which the FRP confines the growth of concrete cracks and delays the loss of the aggregate interlock. The shear strength of reinforced concrete members strengthened with externally bonded fiber-reinforced polymer (EB-FRP) is calculated, in all the design codes, by the linear superposition of the shear strength contributions of FRP, $V_{FRP}$, concrete, $V_{c}$, and steel, $V_{s}$.

Most of the published body of research considered the problem of strengthening of concrete members with FRP for flexure. Over time, this has led to the development of reliable methods for the design and analysis of such structures. Design guidelines have also been developed to incorporate some of the most accurate methods, the list includes ACI PRC-440.2-17 [1] and European *fib*-TG9.3 [2]. However, such research and development have not been translated to the case of concrete members strengthened to resist shear loads, in which the body of experimental studies is very limited, and the developed theoretical models lack the necessary accuracy to be used in design and analysis.

The behavior of shear-strengthened RC members is complicated and is often affected by several parameters and variables, such as the member geometry, flexural and shear reinforcements, the FRP type and fiber orientation, etc. [3,4]. Other parameters that add to the already complex shear behavior of strengthened RC members include the possible interaction between steel shear reinforcement and FRP composite [5].

The shear strengthening of RC members with EB-FRP remains a current research problem and requires more investigations to be completely understood. Examples of recent research studies on this topic include Bousselham and Chaallal [6], Lu et al. [7], Mofidi and Chaallal [8], Nguyen-Minh and Rovnak [9], and Karzad et al. [5]. Except for Karzad et al. [5], past experimental studies focused mainly on exploring the effectiveness of EB-FRP shear strengthening techniques and configurations. Moreover, the previously proposed predictive models, when compared to test results are found to be less accurate [10], which indicates that other parameters that may influence the EB-FRP shear resistance have not yet been determined, and many interactions between the parameters have still not been fully discovered.

The effects of the transverse steel reinforcement on the FRP efficiency have been studied by Deniaud and Cheng [11]. The authors tested eight reinforced concrete beams with different ratios of shear reinforcement and the obtained results showed that high amounts of the steel stirrups reduce the efficiency of FRP strengthening. Three year later, Bousselham and Chaallal Bousselham and Chaallal [3] tested 12 FRP-strengthened beams to investigate the interaction between the FRP and the stirrups in terms of shear gain. The authors found that in slender beams, the increase in stirrup reinforcement ratio sharply decreases the shear gain. However, the transverse steel reinforcement does not affect the shear gain in deeper beams.

Perhaps a more complete study on the present topic is the one reported by Mofidi and Chaallal [4]. In this study, the effects of various parameters influencing the shear behavior of EB-FRP beams was discussed and the accuracy of the current guidelines was assessed through laboratory tests on full-scale specimens. Their study led them to propose a new design model that takes into consideration the effects of transverse steel reinforcement, which was found to have a significant effect on the shear capacity of strengthened beams. Three years later, the authors studied the effects of the transverse steel reinforcement on the shear gain by testing ten CFRP-strengthened T-beams [12]. They considered several design parameters, including the transverse steel ratio. It was observed that increasing the steel shear reinforcement ratio decreases the gain in shear capacity due to the contribution of FRP and that both ACI PRC-440.2-17 [1] and HB 305-08 [13] failed to capture this phenomenon. In a recent study carried by Karzad et al. [5], the interaction between conventional shear reinforcement and the EB-CFRP strips has been studied by testing 16 shear-strengthened and repaired rectangular RC beams with two different shear reinforcement ratios. The authors demonstrated that the contribution of CFRP in shear strength is affected by the quantity of steel stirrups and that when compared to test results, the ACI PRC-440.2-17 and European *fib*-TG9.3 formulas provided reasonable estimation of the additional shear strength contributed by the EB-CFRP discrete strips, except for the beams with moderate ratio of steel stirrups.

Despite the findings of previous and recent experimental studies, focus has mainly been given to exploring the effectiveness of EB-FRP shear strengthening systems and configurations. In addition, other parameters that may influence the EB-FRP shear resistance have not yet been determined, and many interactions between the parameters have still not been fully discovered. The complexity of the shear behavior of RC beams is high and is further increased owing to the presence of FRP. Therefore, the present work attempts to:

1. Prepare 11 full-scale RC T-beams with and without EB-FRP and test them in the laboratory.
2. Investigate the interaction between steel stirrups and EB-FRP strips and the influence of this interaction on the shear carrying capacity of strengthened RC T-beams. This is accomplished considering the following main parameters:
  - the EB-FRP and steel stirrup reinforcement ratios;
  - the location EB-FRP strips with respect to the steel stirrups.
3. Use the test data to assess performance of the ACI and fib predictive models.

## 2. Summary of Related Studies

In addition to the studies highlighted earlier in the introduction, several other researchers have contributed to the experimental studies of the shear strength of EB-FRP. This section provides a summary of the main results of the most relevant of such studies, including those that focused on dealing with the issue of interaction between FRP and shear reinforcement.

Strengthening and repair of reinforced concrete structures can be achieved through different methods and techniques, such as prestressing, shotcreting, and steel plate bonding (Bournas et al. [14], Al-Mahaidi and Kalfat [15], Siddika et al. [16]). An alternative to such methods is the use of externally bonded fiber-reinforced polymer reinforcement. FRP materials possess several advantages over other strengthening materials, such as high tensile strength, high stiffness, corrosive resistance, low thermal transmissibility, as well as ease of application making them suitable for the use as concrete structural reinforcement (Huo et al. [17], Ferdous et al. [18]). Compared to conventional strengthening techniques, FRP strengthening of reinforced concrete members results in superior performance (Fathelbab et al. [19]).

The carbon fiber-reinforced polymers (CFRP), the glass fiber-reinforced polymers (GFRP), or the aramid fiber-reinforced polymers (AFRP) are the three types of FRP, combined by resin and additives, formatting the polymer matrix (Mugahed et al. [20]).

Externally bonded FRP reinforcements can be bonded in S, U or fully wrapped configurations to shear strengthen the reinforced concrete beams (Siddika et al. [16]). Moreover, FRP can be discrete in the form of strips or continuous in the form of sheets, and the fibers can be oriented vertically or diagonally. The maximum tensile strength of the fibers can be obtained when the FRP is oriented in a way that the principal fiber direction is parallel to the direction of the highest principle tensile stresses, i.e., when the fibers are oriented at angle 45° with the member axis. For a comprehensive review of past experimental studies involving the shear capacity of RC beams strengthened with EB-FRP, the reader is referred to Kotynia et al. [21].

The performance of the FRP in shear strengthening of concrete structures depends on the interaction between the FRP layers and the steel stirrups. For instance, Salah et al. [22] found that the interaction between the FRP layers and the internal steel stirrups is such that with use of externally bonded FRP, the stirrups contribution to the total shear capacity in the retrofitted beams is reduced. Several researchers have investigated this interaction to provide clear understanding on the FRP contribution in the presence of the steel reinforcement of reinforced concrete beams (Pellegrino and Modena [23], Chen et al. [24], Chen et al. [25], Mofidi and Chaallal Mofidi and Chaallal [8]).

A recent study carried out by Samb et al. [26] compared the effectiveness of shear strengthening with multi CFRP layers, with the performance of a single CFRP layer, and investigated the maximum number of externally bonded CFRP layers that can be installed without exhibiting slippage or debonding. The results of the study showed that the increase in the CFRP weight enhanced the shear resistance up to an optimal value. However, the results of this study showed that as the number of layers increase, the strain reading in the CFRP strips reduce.

## 3. Experimental Program

### 3.1. Test Specimens

**Figure 1:**
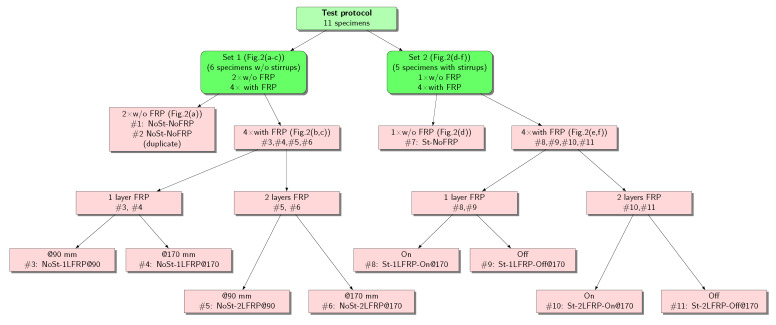
Description of test specimens.

To achieve the objectives set for this research, a total of 11 RC beams were prepared and properly instrumented. The 11 beams were divided into two sets based on the presence of shear reinforcement and/or FRP strips. Set 1 consists of six beams without shear stirrups but strengthened using one or two layers of FRP strips with 90- and 170-mm c/c spacing, and is designated as NoSt-nLFRP@s. Two beams of this set, designated as NoSt-NoFRP, were not strengthened with FRP strips and ware used as a control beam for this group. Set 2 consists of five beams with shear stirrups with a spacing of 170 mm c/c. One beam of this set, designated as St-NoFRP, was not strengthened with FRP strips and was used as a control beam for this group. The remaining four beams were strengthened using one or two layers of FRP strips with a 170 mm c/c spacing at two different locations relative to the transverse shear stirrups (i.e., between two stirrups and on the steel stirrups), and are designated as St-nLFRP-ON or St-nLFRP-OFF. The flowchart in Figure 1 describes the two experimental sets.

**Figure 2:**
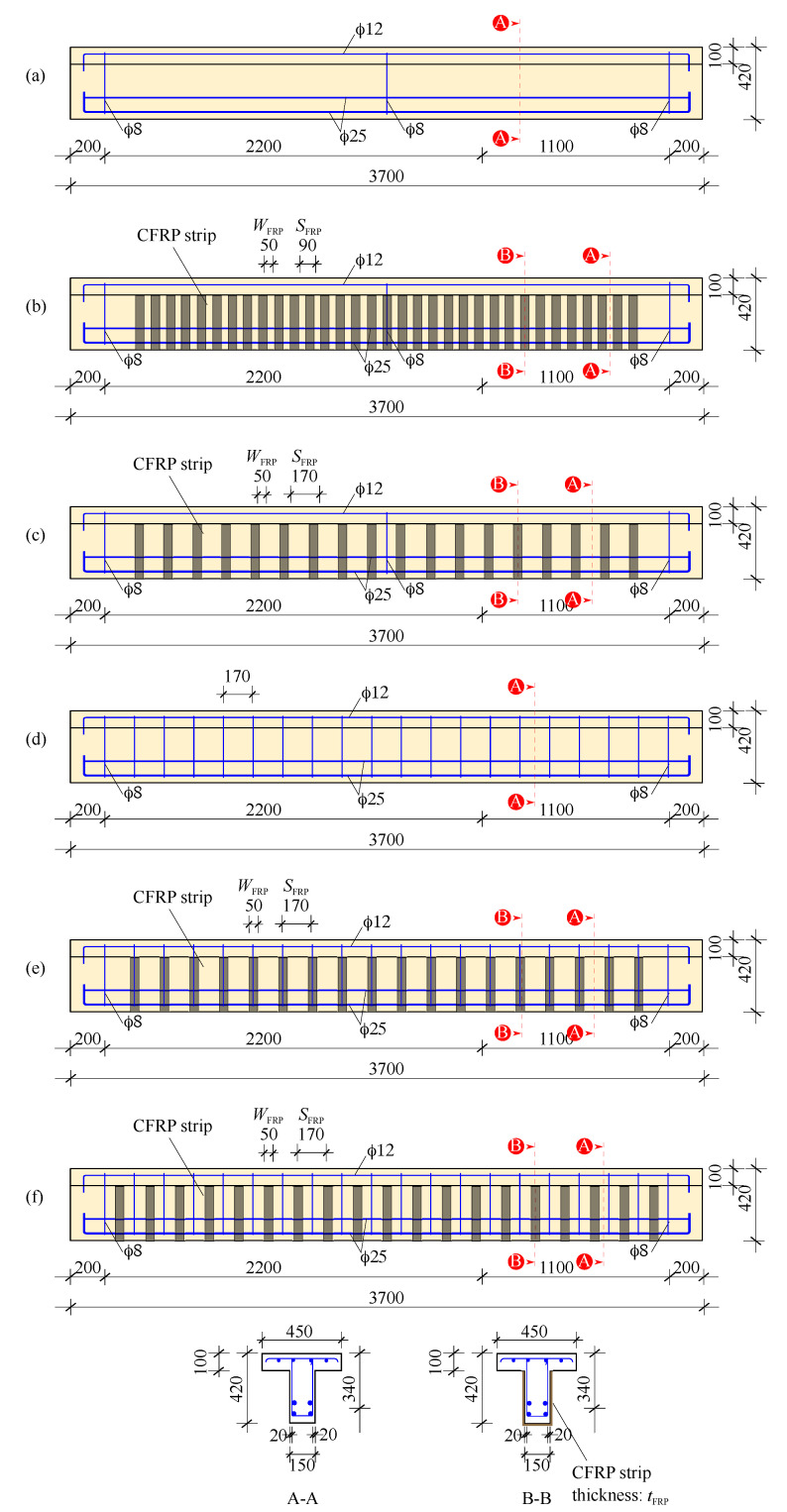
Geometry of test specimens and reinforcement detailing (all units are mm); (**a**) NoSt-NoFRP, (**b**) NoSt-FRP@90, (**c**) NoSt-FRP@170, (**d**) St-NoFRP, (**e**) St-FRP-On, and (**f**) St-FRP-Off.

Figure 1 and Figure 2 illustrate the geometry and reinforcement detailing of test specimens. All specimens have a T-section with a web width of $b_{w}=150$ mm, flange width of $b_{f}=450$ mm, flange height of $h_{f}=100$ mm, total height of h=420 mm, effective depth of d=340 mm, length of L=3700 mm, and test span = 3300 mm. To ensure that shear is the dominant mode of failure, all beam specimens were over reinforced with 4ϕ25 longitudinal steel bars in the tension zone and 4ϕ12 bars in the compression zone. For shear reinforcement, mild steel closed stirrups of 8 mm in diameter were used. In the case of beam shear strengthening, one or two layers of EB-FRP discrete strips in a U-jacketing configuration were used. Table 1 reports the designation and properties of test specimens.It should be noted that the ACI-318-19 code was referred to for the design of conventional steel shear reinforcement while the ACI PRC-440.2-17 guide was referred to for the design of FRP.

### 3.2. Materials

#### 3.2.1. Concrete

Table 2 reports the mix proportions of concrete used to cast the RC beam specimens. The concrete mix comprised type I Portland cement, coarse aggregate with a maximum size of 20 mm and fine aggregates were composed of both washed and dune sand. To enhance the workability of concrete, “SP700” was used as a superplasticizer. As defined by the manufacturer, *"the SikaPlast-700, or SP700, in short is a polycarboxylate-based superplasticizer developed particularly for use in ready mixed concrete to give extended slump retention and high-strength development of normal grade concrete mixes"*. When it meets the cement particle, the SP700 generates electrostatic repulsion resulting in a higher dispersion, flow, and retention. The average compressive strength, $f_{cu}$, of the mix was determined experimentally, after 28 days as per ASTM C39 [27], to be about 68 MPa.

#### 3.2.2. Steel Reinforcement

For the longitudinal reinforcement bars, ϕ25 mm and ϕ12 mm with an average yield strength of $f_{y}=559$ MPa and $f_{y}=575$ MPa were used to reinforce the tension and compression zones, respectively. For the shear reinforcement ϕ8 mm bars with $f_{y}=559$ MPa were used. The modulus of elasticity of all steel bars (longitudinal and transverse) is $E_{s}=200$ GPa.

#### 3.2.3. CFRP and Resins

The EB-CFRP sheets used were unidirectional Carbon Fiber-Reinforced Polymer (CFRP). The mechanical properties of these sheets as reported by the manufacturer are elastic modulus of CFRP $E_{CFRP}=230$ GPa, and ultimate elongation $\epsilon_{CFRP}=180$%.

Before the application of EB-CFRP, the surface of each beam specimens was prepared using Nitomortar FC™, which is a special-purpose mortar used in leveling and filling of small gaps in concrete. Prior to bonding the CFRP discrete strips, a compatible primer resin commercially called Nitowrap FC™ was used for both sides and soffit of the beams. The bonding and encapsulation of EB-CFRP sheet was performed using a compatible epoxy resin. This resin has a flexural strength greater than 40 MPa, flexural modulus greater than 3500 MPa, and adhesion strength greater than 2 MPa.

### 3.3. Test Setup and Instrumentation

**Figure 3:**
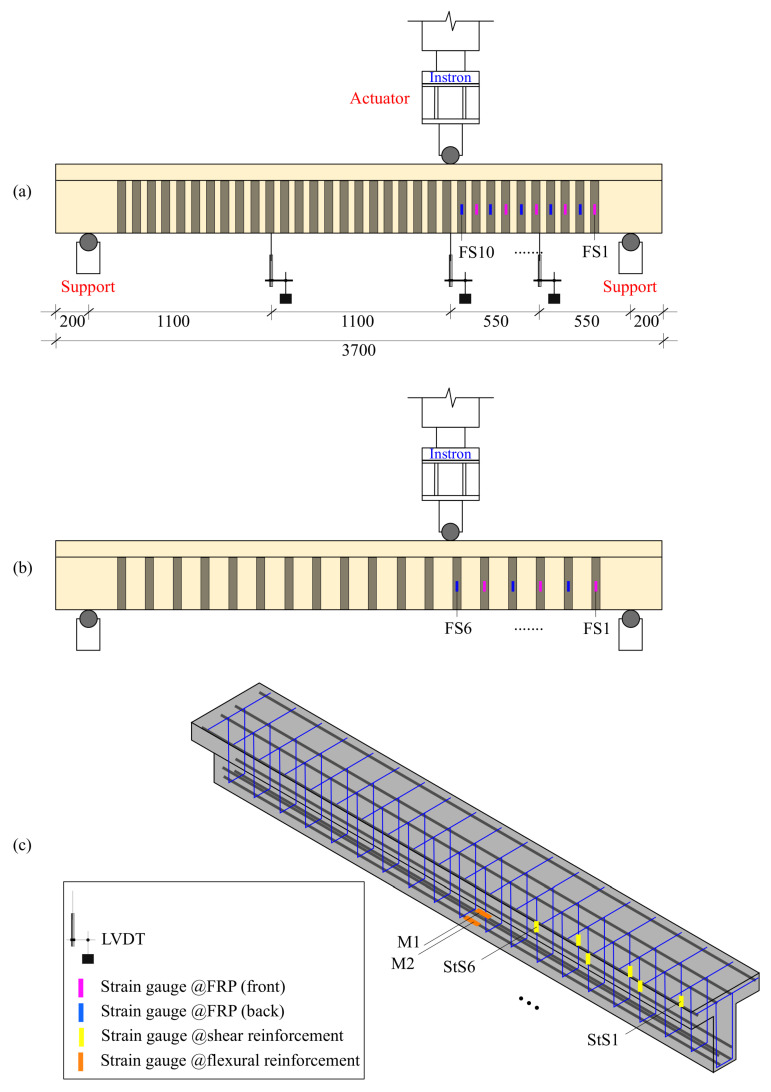
Test setup and instrumentation: (**a**) instrumentation of the beam with 90-mm spaced FRP strips; (**b**) instrumentation of the beam with 170-mm spaced FRP strips; (**c**) instrumentation of steel bars and stirrups (all units in [mm]).

**Figure 4:**
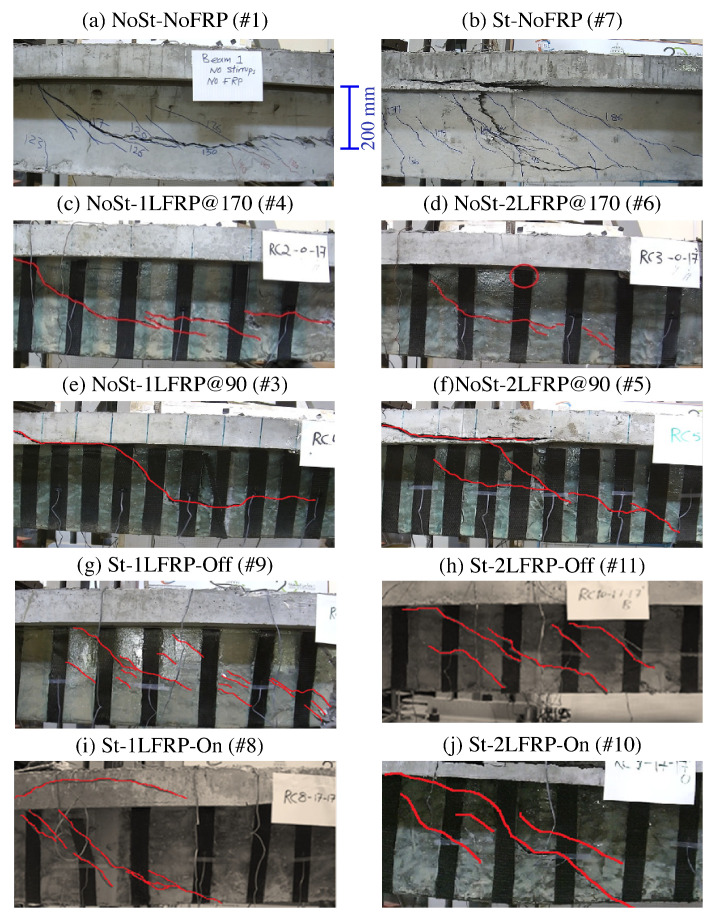
Failure modes of tested specimens.

The experimental setup consisted of three-point loading configuration by placing each beam specimen on two roller supports. The load was applied at a distance of a=1100 mm from the nearest support equal to one third the beam test span (Figure 3). This loading configuration was chosen because it enables the shear failure location to be within a shorter span by increasing the shear force and reducing the maximum bending moment under the load. The loading was applied using an Instron^®^ actuator featuring a capacity of 500 kN for specimen with perceived lower capacity, while for specimens with perceived higher capacity (more than 500 kN) a 1500 kN Dartec^®^ actuator was used instead. The applied load was monotonically increasing in a quasi-static displacement-control mode with a rate of 0.01 mm/s. Each beam specimen was instrumented with three Linear Variable Displacement Transducers (LVDTs) one at one third bottom of the beam (under the loading point) and two in the middle of the right and left shear spans (Figure 4). In addition, two strain gauges were installed on the main reinforcement to measure the strains in the tensile steel only at the load location. The strain gauges on the stirrups were installed along the anticipated plane of the shear crack. In addition, strain gauges were fixed vertically onto the faces, and parallel to the EB-FRP strips at the same location along the same longitudinal axis as the strain gauges on the transverse steel. These strain gauges were attached to every stirrup and FRP strip starting from the point of loading until the nearest support. In this way, the strains in the FRP strips and the corresponding steel stirrups could be conveniently compared during the various loading stages. Cracks were continuously monitored during test.

## 4. Results and Discussion

### 4.1. Failure Modes

All specimens were video-recorded during their testing using a High-Definition video camera. These videos were processed to track the cracking, failure modes, and the sequence of CFRP strips’ debonding of each beam specimen. Still images taken at the end of each test were marked at the crack locations are shown in Figure 4. Next, we will discuss the mode of failure for each specimen and discuss how it differs from the others.

**Figure 5:**
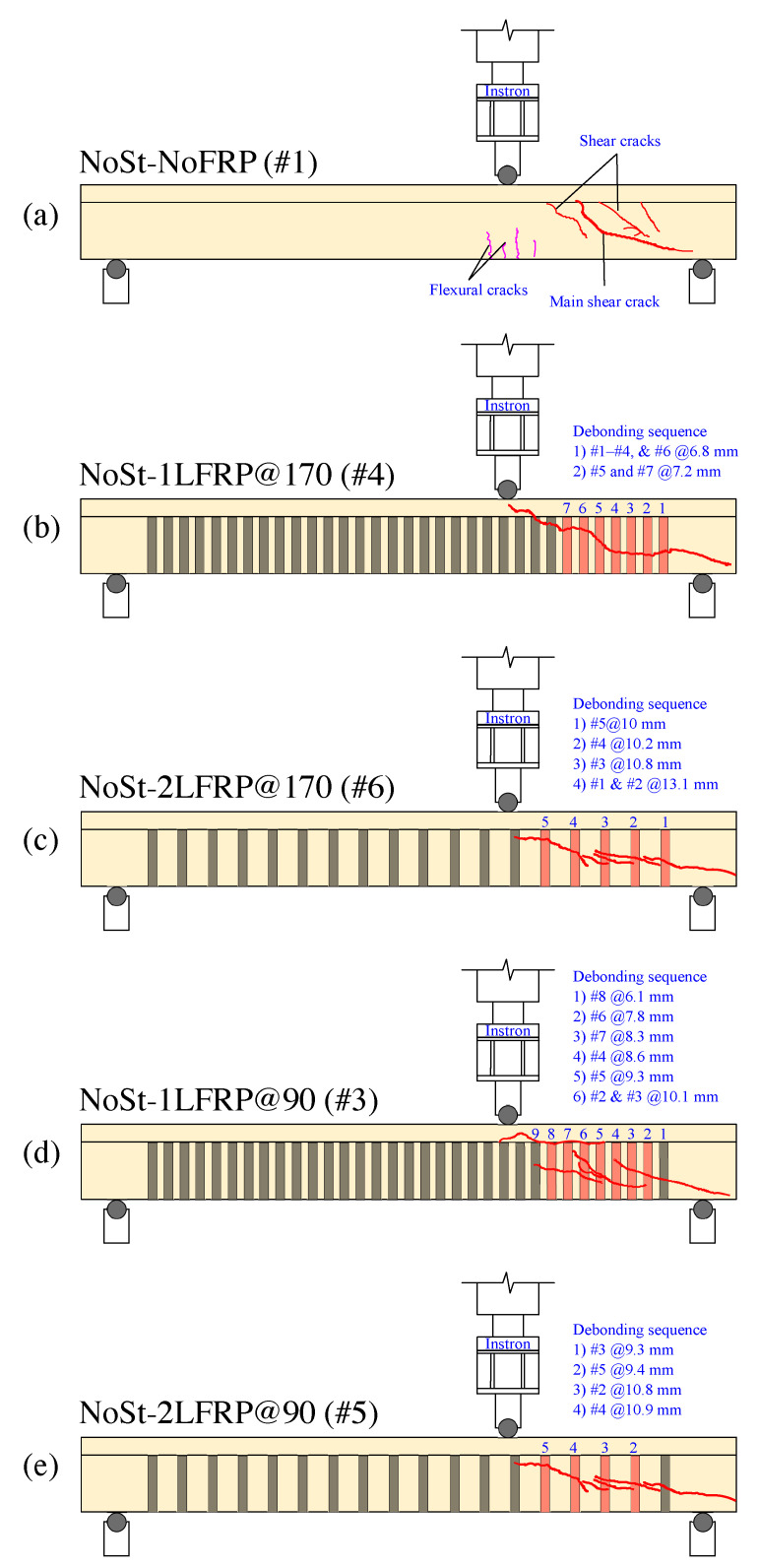
Cracking patterns and FRP debonding sequence (following the deflection @LVDT#2) of specimens without stirrups: (**a**) specimen #1; (**b**) specimen #4; (**c**) specimen #6; (**d**) specimen #3; (**e**) specimen #5.

Figure 5a illustrates the cracking status of the control specimens #1 and #2 (i.e., NoSt-NoFRP) at failure. In these beams, flexural cracks started to appear when the applied load reached about 27 kN, which corresponds to a shear load of 18 kN, at the bottom face of the beam to the right and left of the load application point, then propagated vertically. When the applied load reached about 117 kN, shear cracks started to develop. As the load increased, more shear cracks developed at the short span of the beam (i.e., between the load application point and the right support). Ultimate failure of the beam occurred when a large and long shear crack developed suddenly near the neutral axis in the short span. The failure took place at a load level of 198 kN, which corresponds to a shear force of 130.67 kN. The mode of failure of this beam was of shear type.

Specimens #4 and #6 (NoSt-1LFRP@170 and NoSt-2LFRP@170) experienced almost similar mode of failure, as illustrated in Figure 5b,c. Both specimens developed flexural cracks in the tension zone below the load application point at a load level of about 35 kN. Then, when the applied load reached 130 kN, shear cracks started to appear first to the right of the load application point (i.e., short span region) between CFRP strips 6 and 7 and propagated diagonally until the right support. Finally, both specimens failed when the applied load reached around 257 kN (i.e., shear force of 171 kN). One difference between the response of these two beams was that the NoSt-1LFRP@170 recorded a max deflection of 13.8 mm (LVDT#2 placed below the load application point) whereas NoSt-2LFRP@170 recorded 16.2 mm (LVDT #2). The main difference between these two beams was that the primary diagonal shear crack in NoSt-1LFRP@170 expanded beyond the right support, leading to the crushing of concrete below the support. This behavior has not been observed in the NoSt-2LFRP@170. The difference in the length of the primary shear crack led to the debonding of five CFRP strips (out of the total six strips in the short span) of the NoSt-1LFRP@170 whereas only three strips debonded in the NoSt-2LFRP@170. Further discussion on the debonding of CFRP strips is presented in Section 5 and Section 6.

As illustrated in Figure 5d,e, specimens #3 and #5 (NoSt-1LFRP@90 and NoSt-2LFRP@90) experienced almost the same mode of failure; both developed flexural cracks in the tension zone below the load application point at a load level of about 35 kN. In addition, at a load level of about 130 kN, shear cracks started to appear first to the right of the load application point between CFRP strips 5 and 8 then propagated diagonally until the right support. Moreover, both specimens failed ultimately when the applied load reached around 220 kN, which represents a shear force of about 147 kN. One difference between their responses was that the NoSt-1LFRP@90 recorded a maximum deflection of 6.7 mm (LVDT#2) whereas NoSt-2LFRP@90 recorded 10.2 mm (LVDT #2). One difference between the response of these two beams was that the failure of the NoSt-2LFRP@90 was more progressive, demonstrated by the debonding of one strip at a time. Compared to the previously discussed specimens (NoSt-1LFRP@170 and NoSt-2LFRP@170), the shear cracks in the current specimens expanded up to the flange to the right of the point of load application. This behavior is due mainly to the number of CFRP strips; in the beams with 90 mm-spaced strips (NoSt-1LFRP@90 and NoSt-2LFRP@90), the web became stronger because of the ten CFRP strips, which exposed the top flange as a weak spot susceptible to shear cracking, starting at the surface connecting the web to the top flange.

The crack development mechanism of the beam St-NoFRP (specimen #7) was very similar to the beam NoSt-2LFRP@90 (specimen #5); both failed ultimately following a major diagonal crack that started in the web in the middle of the short span, which propagated to the right support and to the flange up to the load application point. There was, however, one major difference between the two beams before failure; the current beam (St-NoFRP) experienced multiple diagonal and parallel cracks in the short span region. Moreover, the current failed at a load level of 416 kN, which is equivalent to a shear load of about 277 kN, recorded at a deflection of 20 mm under the point of loading.

**Figure 6:**
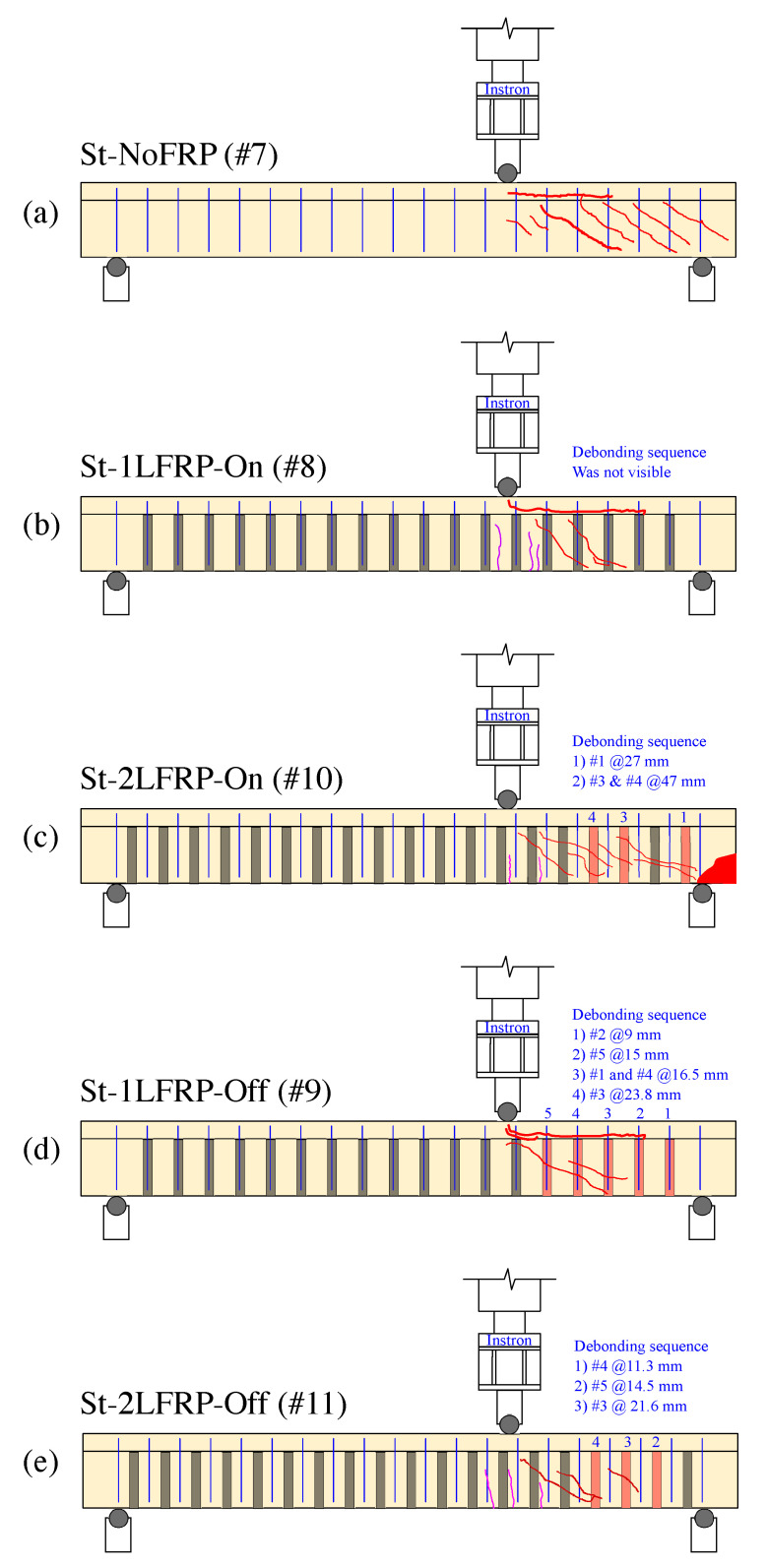
Cracking patterns and FRP debonding sequence (following the deflection @LVDT#2) of specimens with stirrups: (**a**) specimen #7; (**b**) specimen #8; (**c**) specimen #10; (**d**) specimen #9; (**e**) specimen #11.

Specimen #8 (St-1LFRP-On) developed similar crack pattern to Specimen #7 (St-NoFRP), as illustrated in Figure 6a,b and similar ultimate load capacity. The only difference is the ultimate deflection recorded by LVDT2; the current beam failed at a deflection that is twice the deflection at which the beam St-NoFRP failed.

The failure mechanisms of specimen #10 (St-2LFRP-On), shown in Figure 6c, and specimen #8 (St-1LFRP@170) were almost the same from the beginning until reaching the ultimate load capacity of 410 kN. Beyond this point, specimen #10 (St-2LFRP-On) had three CFRP strips debonded, leading to its premature failure and the drop in its load-carrying capacity. Specimen #8 (St-1LFRP-On) continued to maintain its load-carrying capacity for much longer and failed ultimately at a deflection of about 40 mm, recorded by LVDT#2.

Figure 6d illustrates the failure mode of specimen #9 (St-1LFRP-Off). Prior to failure, several cracks appeared on the surface of the beam in the short span, and as the load continued to increase, new cracks appeared, and the previous ones increased in width and depth. One vertical crack developed at the flange right below the point of loading; however, it remained small and narrow. Failure of this beam was mainly due to the multiple cracks and the crushing of the concrete at the right support location. CFRP strips debonding was not noticeable during the test nor prior to failure. The ultimate load was slightly higher than the one achieved by specimen #8 (St-1LFRP-On).

The failure mechanism of specimen #11 (St-2LFRP-Off), illustrated in Figure 6e, was similar to specimen #9 (St-1LFRP-On). However, the cracks in the current specimen were smaller in number and the ultimate failure involved the debonding of at least two strips (4 and 5). In addition, there was no concrete crushing at the support. This beam’s capacity was identical to specimen #9 (St-1LFRP-On).

### 4.2. Load-Deflection Relationship

**Figure 7:**
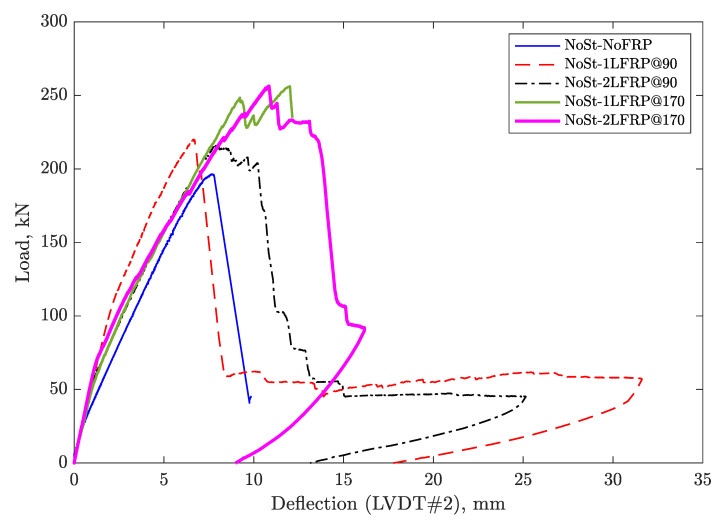
Load versus deflection under point of loading for specimens without stirrups.

**Figure 8:**
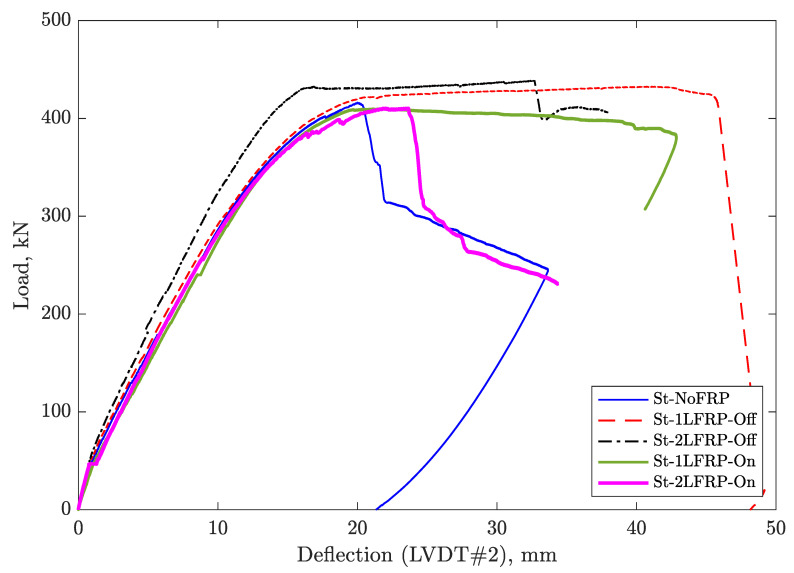
Load versus deflection under point of loading for specimens with stirrups.

Figure 7 and Figure 8 show the curves of the applied load versus the deflection recorded by LVDT#2 for the specimens with and without steel stirrups, respectively. As a first observation, the beams without transverse steel reinforcements failed promptly after reaching their ultimate load capacity, and those strengthened with CFRP, their failure involved the debonding of the some CFRP strips. Furthermore, it was noted that the beams that had no stirrups but only FRP strips at a 90-mm spacing behaved similarly to the unenforced control beam, in the sense of the load drop, which occurred owing to the spontaneous debonding of most of the FRP strips. However, the shear induced crack did not result in immediate rupture of the beam, which held on until the crack widened to the maximum deflection and ultimately failed.

Adding a layer of CFRP strips spaced at 90 mm to the beam without stirrups did not change the overall load-deflection relationship, except about 9% increase in the load capacity. Adding another layer, however, resulted in a relatively more ductile behavior, which was demonstrated through the observed relatively gradual and progressive debonding of nine CFRP strips.

For beams without stirrups, adding a single layer of CFRP strips spaced at 170 mm increased the load-carrying capacity by about 30%, but when spaced at 90 mm, the gain reduced to 12% only. This can be explained by the fact that the NoSt-1LFRP@90 and NoSt-2LFRP@90 have more FRP trips, hence stronger webs, which exposed the top flange as the weakest spot susceptible to shear cracking, starting at the surface connecting the web and top flange. Moreover, adding a second layer of CFRP strips spaced at 170 mm did not result in any improvement.

For unwrapped beams, adding a minimum of shear reinforcement (i.e., stirrups spaced at 170 mm), doubled the load-carrying capacity of the beam. Adding one layer of CFRP strips to the beam with minimum shear reinforcement at the location of the stirrups did not result in any improvement in the load-carrying capacity, even after adding another layer. The only noticeable benefit of adding one layer of CFRP was in the delay of failure as demonstrated by the ultimate displacement. By changing the location of the CFRP strips to in between the stirrups, resulted in a small improvement in terms of the ultimate strength. The stiffness of all tested specimens was the same at about 25 kN/m.

Based on the load-deflection curves, the yield displacement, yield load, and maximum displacement were determined for specimens #8-#11 and reported in Table 3. In addition, the table reports the displacement ductility for each specimen as well. The yielding point at which the yield displacement and yield load were determined correspond to the point at which the first yielding of the longitudinal reinforcement occurred. The maximum displacement reported in the table corresponding to the displacement at which the specimen lost about 20% of its load-carrying capacity. The three displacements reported in the table correspond to the three LVDTs (#1, #2, and #3) and the ductility is calculated for each one of these displacements.

For the two specimens with stirrups and one layer of CFRP (i.e., specimens #8 and 9), the data shows that the yielding of longitudinal reinforcement occurred almost at the same load level ($P_{y}$). However, the deflection at failure and the displacement ductility of specimen #8 were 6–16% and 5–9% smaller than specimen #9, respectively, depending on the recording LVDT.

For the two specimens with stirrups and two layers of CFRP (i.e., specimens #10 and #11), the results show that there is no difference between the two beams in terms of the load capacity at yielding ($P_{y}$). However, the deflection at failure, $d_{m}$, and the ductility, μ, were substantially higher in the beam with CFRP layers placed between the stirrups (i.e., specimen #11). The difference amounts to two times especially when the deflection recorded by LVDT1 is considered.

Although the two beams (St-2LFRP-On and St-2LFRP-Off) have had the same load-carrying capacity, their ultimate failure was different; the failure in the St-2LFRP-Off-beam toke place at larger deflection compared to the St-2LFRP-On. This is a desirable behavior often sought by designers to avoid sudden catastrophic failures; ductile beams fail in a progressive fashion.

## 5. Strain Response

In this section, the strain response is discussed for each specimen with CFRP. The reported strains pertain to those recorded by the strain gauges placed on the longitudinal reinforcements and those on the CFRP strips. We start first by discussing the beams without shear reinforcement (Specimens 3, 4, 5, and 6) then the beams with shear reinforcement (Specimens 8, 9, 10, and 11).

### 5.1. Specimens with CFRP and without Shear Reinforcement

**Figure 9:**
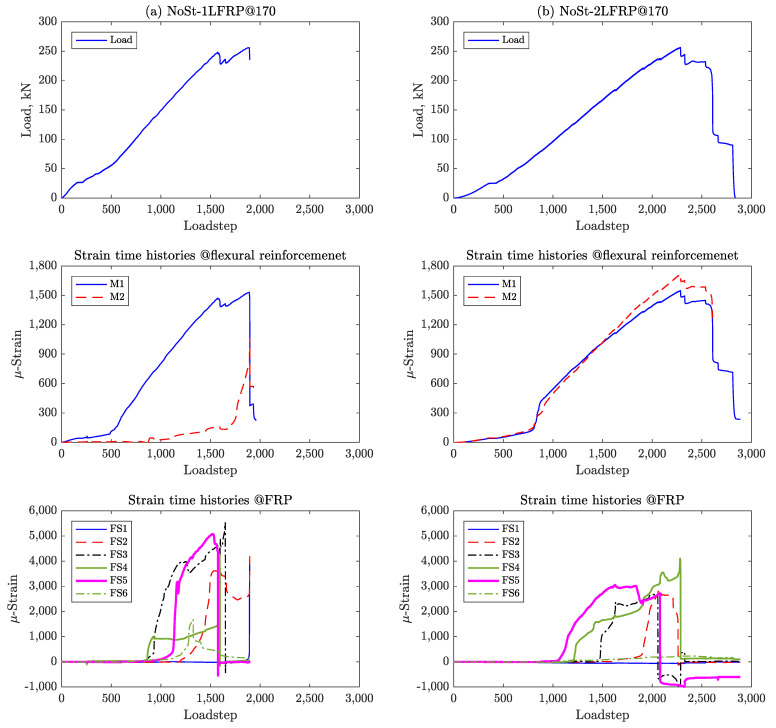
Strain time histories for (**a**) NoSt-1LFRP@170 and (**b**) NoSt-2LFRP@170.

**Figure 10:**
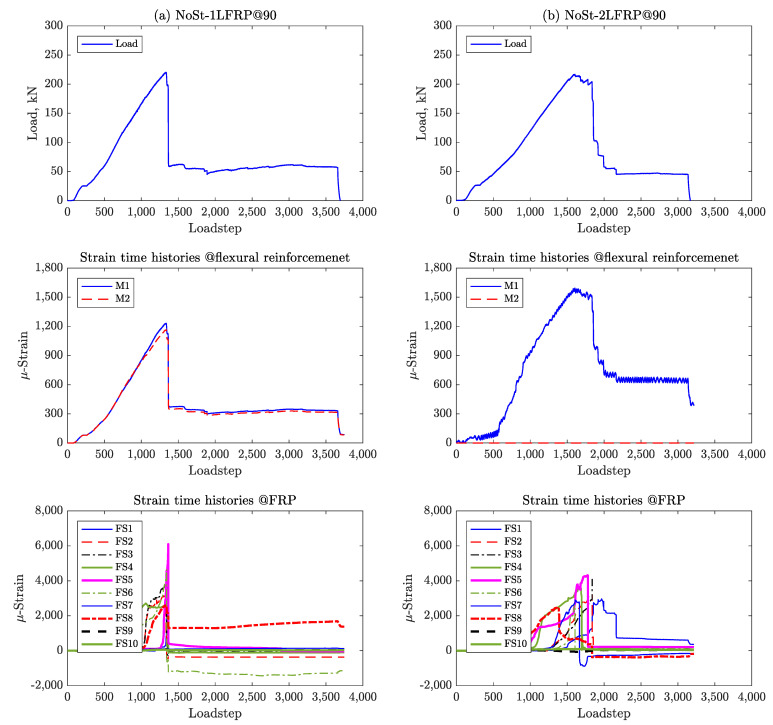
Strain time histories for (**a**) NoSt-1LFRP@90 and (**b**) NoSt-2LFRP@90.

Figure 9 and Figure 10 to show the load and CFRP strain pseudo-time histories for all the specimens plotted at each pseudo-time step, which corresponds to a displacement increment of 0.01 mm.

Specimen #4, which has one layer of CFRP spaced at 170 mm but lacks shear reinforcement (i.e., NoSt-1LFRP@170) was observed to fail in shear and CFRP debonding progressively. This can be confirmed by the plots in Figure 9a; the load-deflection curve shows gradual drops in the load capacity of the beam with each drop corresponding exactly to the moment of debonding of one strip of CFRP as shown in the strain pseudo-time histories. The progressive debonding of the CFRP strips started with strip 5, then 4, all the way to strip 1, which is the closest to the right support. Please note that this beam failed before yielding of the flexural reinforcement as demonstrated by the readings of strain gauges M1 and M2 shown. The same beam but with two layers of CFRP strips (Specimen #6, NoSt-2LFRP@170) experienced similar strain response and progressive debonding behavior. The only difference was that the two strips closest to the load application point (strip 6) and the right support (strip 1) did not debond even after the failure of the beam (see Figure 9b).

Specimen #5 (NoSt-2LFRP@90), which features two layers of CFRP spaced at 90 mm, experienced short but progressive debonding of its CFRP strips, much like the previously discussed beams. However, when only one layer is applied (Specimen #3, NoSt-1LFRP@90), all CFRP strips debonded simultaneously at the peak of the beam’s capacity, except, strip 4 which debonded after failure. The observed debonding mechanism is clearly demonstrated by the sharp drop in the strains recorded by the strain gauges placed on the CFRP strips (Figure 10). Again, for these two beams, the longitudinal reinforcements did not yield.

### 5.2. Specimens with CFRP and Shear Reinforcement

**Figure 11:**
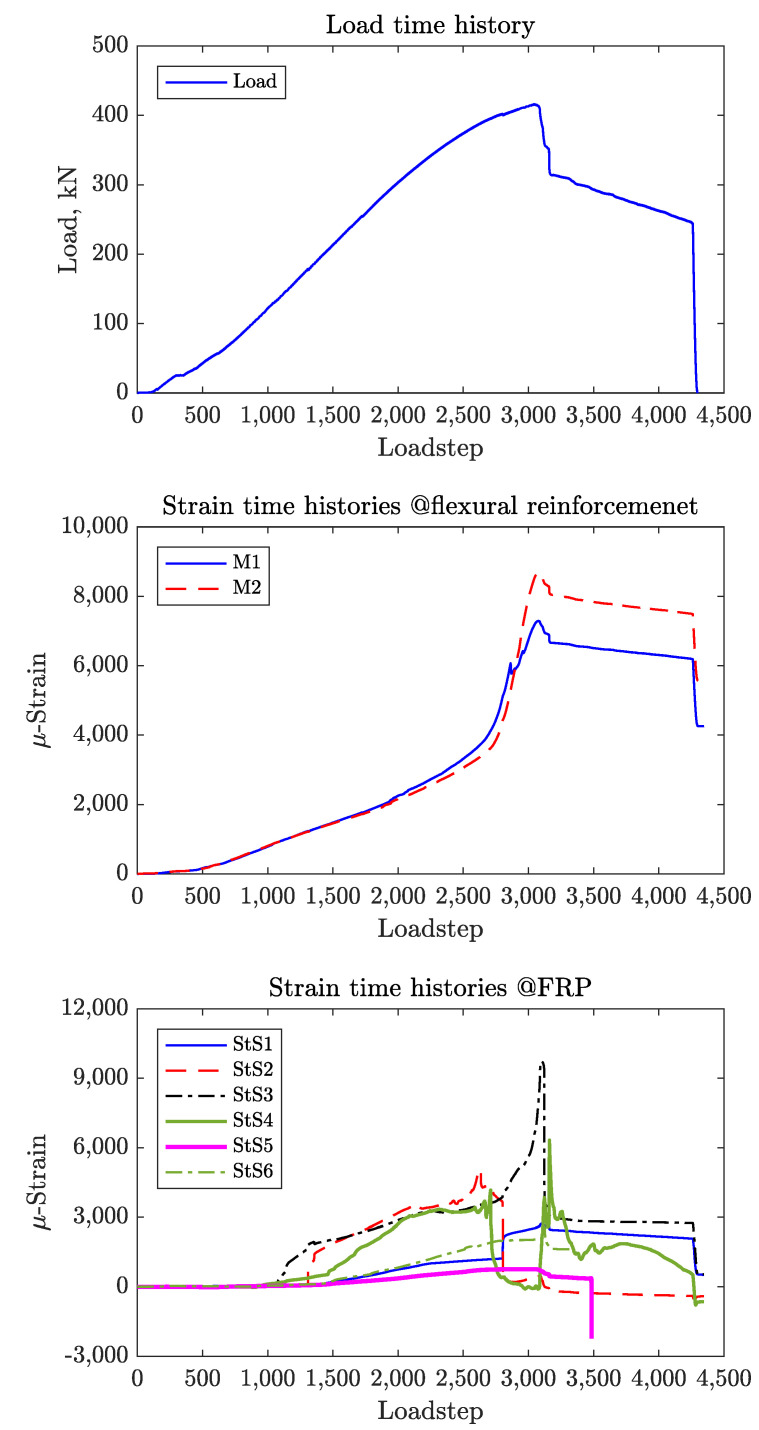
Strain time histories for St-NoFRP.

**Figure 12:**
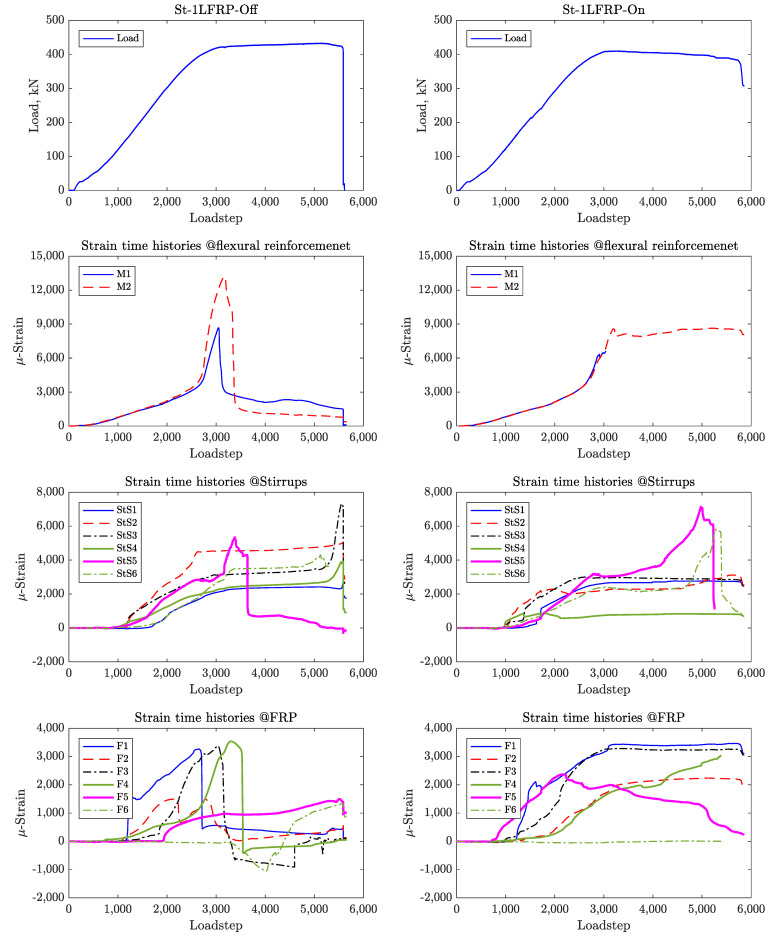
Strain time histories for St-1LFRP-Off and St-1LFRP-On.

**Figure 13:**
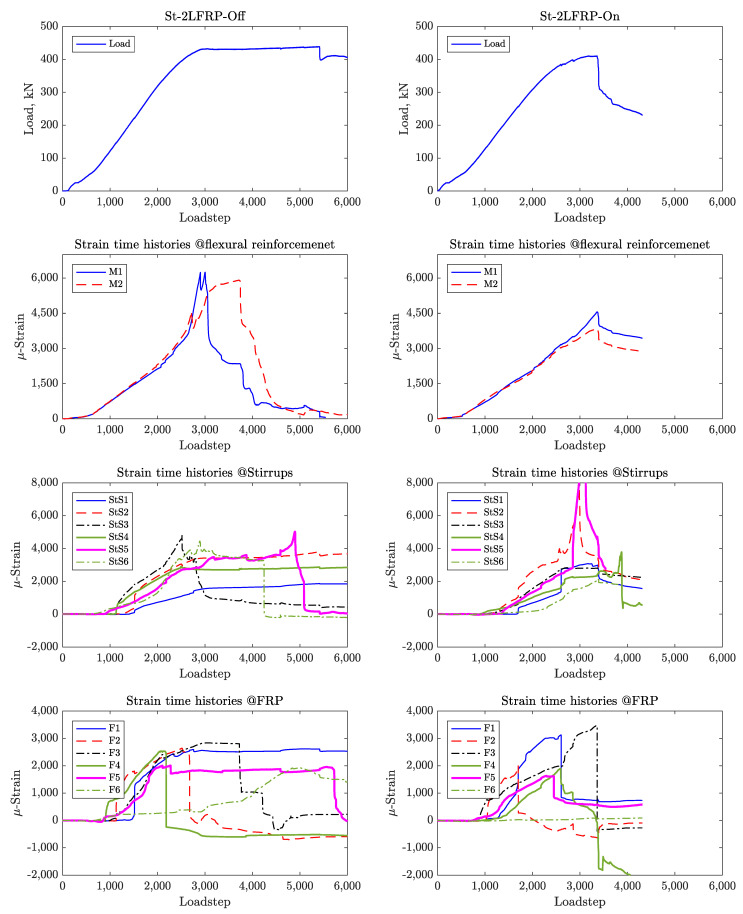
Strain time histories for St-2LFRP-Off and St-2LFRP-On.

For these specimens, there were six strain gauges placed on the steel stirrups in addition to those placed on the CFRP strips and longitudinal reinforcement. For comparison, Figure 11 shows the strain time histories for St-NoFRP while Figure 12 and Figure 13 show the strain time histories for the four specimens St-1LFRP-On, St-1LFRP-Off, St-2LFRP-On, and St-2LFRP-Off, respectively.

For the specimens St-1LFRP-Off and St-1LFRP-On specimens, as shown in Figure 12, the strain activity in the stirrups started at a load level of about 130 kN, which corresponds to the onset of development of shear cracks. Compared to the St-1LFRP-On, these strains continued to increase in the St-1LFRP-Off until the specimen’s failure. Moreover, four steel stirrups in the St-1LFRP-Off-beam (Stirrups 2, 3, 5, and 6) yielded before the beam reached its ultimate load capacity, whereas only two stirrups (Stirrups 3 and 5) of the St-1LFRP-On-beam yielded by the time the beam reached its ultimate capacity. Judging by the strain activity of CFRP in St-1LFRP-On, all strips did not debond even after the failure of the specimen. However, three CFRP strips debonded in the St-1LFRP-Off specimen by the time it reached its ultimate capacity.

**Figure 14:**
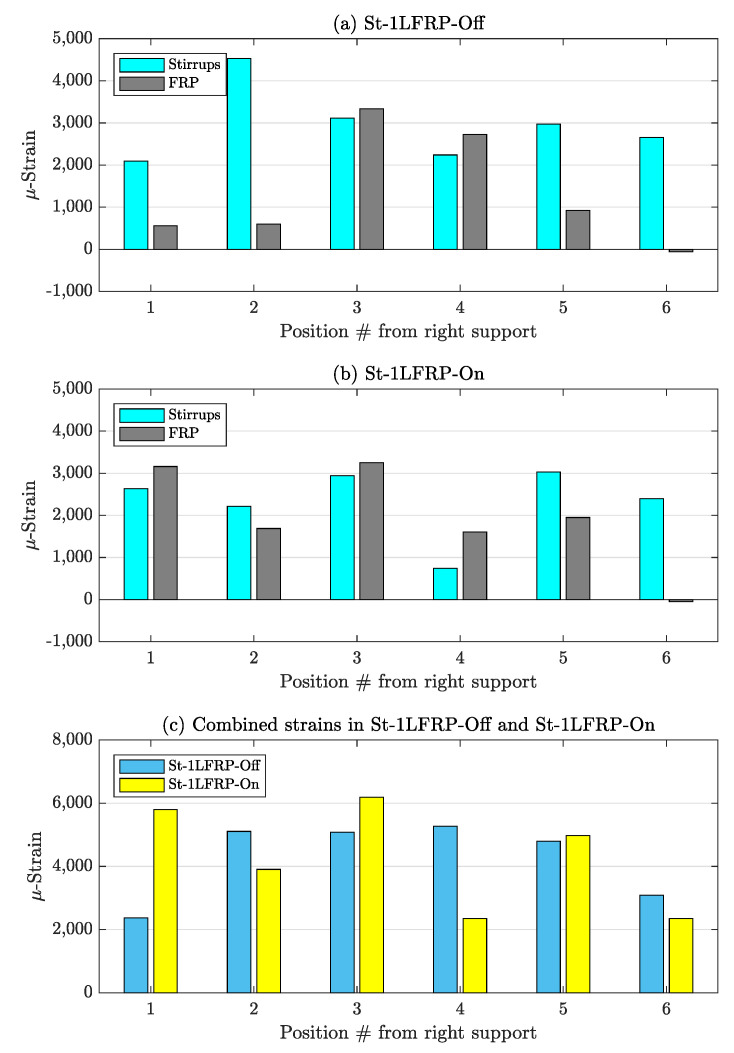
Strain in stirrups and CFRP strips at the load level corresponding to the development of major shear crack: (**a**,**b**) strain in individual stirrups and CFRP strips of St-1LFRP-Off and St-1LFRP-On-beams; (**c**) combined strain in both beams.

Figure 14 shows the strain in the stirrups and CFRP strips and the combined strain (i.e., strain added together) at the load corresponding to the development of the major shear crack (about 418 kN) in St-1LFRP-Off and St-1LFRP-On specimens. The figure demonstrates that overall, both beams were strained almost the same. However, these strain levels are not shared equally between the stirrups and CFRP strips. By the time the St-1LFRP-Off-beam developed a major shear crack, the stirrups recorded higher strain levels than the CFRP, which indicates that their contribution to the overall shear capacity of the beam is higher than in the case of the St-1LFRP-On-beam. The stirrups and CFRP strips in the St-1LFRP-On-beam have almost equal shares of the strain. Please note that the above results were verified to be consistent at all load levels, at least before the debonding of CFRP strips.

**Figure 15:**
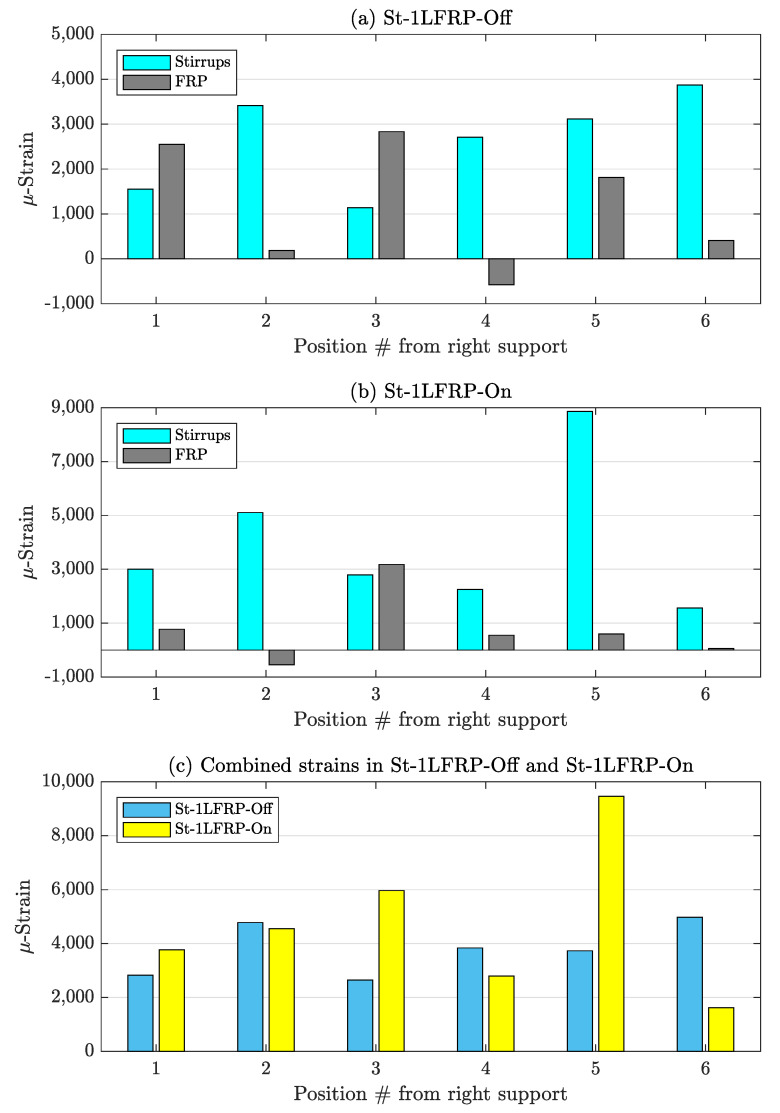
Strain in stirrups and CFRP strips at the load level corresponding to the development of major shear crack: (**a**,**b**) strain in individual stirrups and CFRP strips of St-2LFRP-Off and St-2LFRP-On-beams; (**c**) combined strain in both beams.

For the beams with two layers of CFRP (Figure 15), the strain levels in the stirrups were higher than those in the CFRP strips. However, when combined, both beams (On and Off) have the same strain level.

In the Off-beams (St-1LFRP-Off and St-2LFRP-Off), the strain levels recorded by the strain gauges of the stirrups of the one-layer specimen are higher than those in the two-layer specimen. The strains in the CFRP of the one-layer specimen were lower than those in the two-layer specimen, most of the time. This means that adding another layer of CFRP relieves the stirrups of the Off-beam from carrying much stress. The opposite is true for the On-beams (St-1LFRP-On and St-2LFRP-On), where adding another layer of CFRP puts the stirrups under much more stress.

## 6. CFRP Contribution and Interaction

The interaction between the steel stirrups and CFRP strips and their contribution to the overall behavior and shear capacity of test specimens were demonstrated through test results and are better explained using the strain data recorded during the tests. Therefore, in this section, we highlight the major observations explaining the contribution of CFRP layers and their interaction with steel stirrups.

It has been shown that both single-layer CFRP-strengthened specimens, placed at the same level with the steel stirrups (i.e., On-beam) and in between the steel stirrups (i.e., Off-beam), were strained almost the same. However, these strain levels are not shared equally between the steel stirrups and CFRP strips. In the beam On-beam, by the time the major shear crack developed, the stirrups recorded higher strain levels than the CFRP strips, which suggests that the contribution stirrups to the overall capacity of the beam is higher than the contribution of CFRP. In the Off-beam, the contributions of CFRP strips placed between the stirrups to the overall capacity of the beam were almost at the same level with the stirrups.

In the Off-beams with one and two layers of CFRP, the strain levels recorded by the strain gauges of the stirrups of one-layer specimen were higher than those in the two-layer specimen but the strains in the CFRP of the one-layer specimen were lower than those in the two-layer specimen, most of the time. This indicates that adding another layer of CFRP relieves the stirrups of the Off-beam from carrying much stress. The opposite was found to be true for the On-beams with one and two layers of CFRP, where adding another layer of CFRP (i.e., from one to two layers) puts the stirrups under much more stress.

Overall, the stirrups of the Off-beam with one layer of CFRP recorded lower strains than the stirrups of On-beam. Adding another layer of CFRP reversed this behavior. In contrast, the strain levels in the CFRP strips of the one-layer Off-beam were higher than those in the On-beam, and adding another layer reversed this observation.

## 7. Verification of Code Models

Table 4 compares the ultimate load-carrying capacity of the specimens, tested as part of the current experiment program, to the predictions, $P_{ACI}$ and $P_{fib}$ of two design codes, ACI [1] and *fib* [2], respectively. The table shows the code-predicted individual contributions of concrete, $V_{c}$, steel stirrups, $V_{s}$, and CFRP, $V_{FRP}$, to the shear capacity of the beams. The average and the coefficient of variation (COV) of code predictions are also shown in the table.

Clearly, the predictions of code-based equations are less than test results, which confirms that both codes are too conservative, hence, the call for much needed modifications, as we will demonstrate next.

Comparing the accuracy of ACI and *fib* predictions for the beams reinforced with CFRP only (i.e., without steel stirrups), ACI performed slightly better when the CFRP strips are spaced at 90 mm, and *fib* performed better when the CFRP strips are widely spaced (170 mm). Moreover, fib performed better for beams reinforced with one layer of CFRP. For the beams reinforced with both steel stirrups and CFRP, the ACI predictions were slightly better than *fib*’s when the beam is reinforced with two layers of CFRP. However, the performance of both codes is almost the same when only one layer of CFRP is used.

An interesting observation can be deduced from the table: predictions of both codes were better for the On-beams (with one and two layers of CFRP) and for the beams with two layers of CFRP (On or Off).

Furthermore, both codes are conservative with an almost equal average of 0.7; however, ACI’s predictions were more dispersed than *fib*’s, as demonstrated by their COVs. Overall, their predictions are not affected by the existence or absence of stirrups and their predictions are the highest when the beams are reinforced with two layers of CFRP, especially in the On-beams with the minimum of steel stirrups.

Both design codes assume a linear relation between the contributions of concrete, steel stirrups and CFRP, which for sure simplifies the calculations and avoids iterative solution schemes. Test results reported in the current and previous studies proved that this relationship is nonlinear. Partly, this nonlinearity has been demonstrated here by the observed interaction between the stirrups and CFRP.

Both ACI and *fib* codes seem to ignore the effect of debonding, which, being the major contributor to the failure of tested specimens, is a critical drawback that should be addressed. In addition, both codes anticipate an increase in the load-carrying capacity of beams when the spacing between CFRP strips is reduced to 90 mm. This contrasts with the present test results, where the beams with closely attached CFRP strips (@90 mm) behaved differently than the beams with strips @170 mm, resulting in a reduced load-carrying capacity than the code predictions. Therefore, future code revisions should address the effects of the width of CFRP strips and their spacings when computing the minimum boundaries for center-to-center spacing values.

Finally, the predictive models by current design codes (not restricted to ACI and *fib*) do not take into consideration the position of CFRP strips relative to the steel stirrups (On or Off). As discussed earlier, the current test results demonstrated that these beams can have different load-carrying capacities that depend on the position of the CFRP strips relative to the position of steel stirrups.

## 8. Summary and Conclusions

In this paper, we presented results of an experimental study on the shear behavior of full-scale RC beams strengthened with CFRP. The experimental program involved testing 11 full-scale RC beams divided into two sets based on the presence of shear reinforcement and/or FRP strips. Set 1 consisted of six beams without shear stirrups but strengthened using one or two layers of FRP strips spaced at 90 and 170 mm. Two control beams of this set were not strengthened. Set 2, on the other hand, consisted of five beams with shear stirrups (spaced at 170 mm). One control beam of this set was not strengthened while the remaining four were strengthened using one or two layers of FRP strips spaced at 170 mm and placed at two different locations relative to the transverse shear stirrups (i.e., either between two consecutive stirrups, Off, or at the same level of stirrups, On). Results of the experimental program were then used to assess the accuracy and performance of the predictions of the two design codes: ACI and *fib*. The investigation reported here led to draw the following conclusions:

1. Strengthening the beams without stirrups with one layer of 90 mm-spaced CFRP strips resulted in a non-influential improvement of the load capacity of the beam. Adding another layer of CFRP did not increase the capacity but improved the beam’s ductility.
2. Strengthening the beams without stirrups with one layer of 170 mm-spaced CFRP strips increased the load capacity of the beams by about 30%. Adding another layer of CFRP did not result in any noticeable improvement in behavior.
3. Adding one layer of CFRP strips to the beam with minimum shear reinforcement at the location of the stirrups (i.e., On) did not result in any improvement in the load-carrying capacity, even after adding another layer.
4. Changing the location of the CFRP strips to in between the location of stirrups (i.e., Off), resulted in a small improvement in terms of load-carrying capacity; however, it significantly improved the ductility of the beams and delayed their failure under the monotonically increasing load.
5. When compared to test results, both design codes (ACI and *fib*) were found to be conservative with an average prediction-to-test ratio of 0.7. In addition, ACI’s predictions were more dispersed than *fib*’s.
6. Overall, predictions by both codes were not affected by the existence or absence of stirrups and that these predictions are the highest when the beams are reinforced with two layers of CFRP, especially in the On-beams with the minimum of steel stirrups.
7. Both design codes seem to ignore the effect of debonding, which, being the major contributor to the failure of tested specimens, is a critical drawback that should be addressed.
8. Both codes anticipate an increase in the load-carrying capacity of beams when the spacing between CFRP strips is reduced to 90 mm. This contrasts with the present test results, where the beams with closely attached CFRP strips (@90 mm) behaved differently than the beams with strips @170 mm, resulting in a reduced load-carrying capacity than the code predictions. Therefore, future code revisions should address the effects of the width of CFRP strips and their spacings when computing the minimum boundaries for center-to-center spacing values.
9. The predictive models by current design codes (not restricted to ACI and *fib*) do not take into consideration the position of CFRP strips relative to the steel stirrups (On or Off). The current test results demonstrated that strengthened RC beams may perform and behave differently depending on the position of the CFRP strips relative to the position of steel stirrups.

## Figures and Tables

**Table 1:** Specimen designations and properties.

Specimen#	Designation	$\rho_{v}$	$\rho_{s}$	$d_{\mathrm{FRP}}$, mm	$t_{\mathrm{FRP}}$, mm	$w_{\mathrm{FRP}}$, mm	$s_{\mathrm{FRP}}$, mm	$\rho_{\mathrm{FRP}}$
1	NoSt-NoFRP	0	0.0385	0	0	0	0	0
2	NoSt-NoFRP	0	0.0385	0	0	0	0	0
3	NoSt-1LFRP@90	0	0.0385	240	0.166	50	90	0.0012
4	NoSt-1LFRP@170	0	0.0385	240	0.166	50	170	0.0007
5	NoSt-2LFRP@90	0	0.0385	240	0.332	50	90	0.0025
6	NoSt-2LFRP@170	0	0.0385	240	0.332	50	170	0.0013
7	St-NoFRP	0.0039	0.0385	0	0	0	0	0
8	St-1LFRP-On	0.0039	0.0385	240	0.332	50	170	0.0007
9	St-1LFRP-Off	0.0039	0.0385	240	0.166	50	170	0.0007
10	St-2LFRP-On	0.0039	0.0385	240	0.332	50	170	0.0013
11	St-2LFRP-Off	0.0039	0.0385	240	0.332	50	170	0.0013

**Table 2:** Concrete mix design.

Components	Quantity, kg/m${}^{3}$
9.5 mm CA	795
19 mm CA	1070
5 mm CA	1215
Sand FA	500
OPC	862
Water	325
SP700 (Superplasticizer)	9.20

**Table 3:** Yielding and ductility properties of Specimens #8–#11.

Specimen	$P_{y}$, kN	$d_{y}$, mm	$d_{m}$, mm	μ
LVDT1				
St-1LFRP-Off	351.0	10.3	27.1	2.6
St-1LFRP-On	345.0	10.1	25.4	2.5
Difference (%)	1.75	1.93	6.85	4.83
St-2LFRP-Off	358.7	13.4	44.37	3.3
St-2LFRP-On	364.9	12.3	17.84	1.4
Difference (%)	−1.71	8.49	148.71	129.26
LVDT2				
St-1LFRP-Off	351.0	13.1	45.8	3.5
St-1LFRP-On	345.0	13.3	42.9	3.2
Difference (%)	1.75	−1.46	6.76	8.34
St-2LFRP-Off	358.7	11.6	32.7	2.8
St-2LFRP-On	364.9	14.7	23.9	1.6
Difference (%)	−1.71	−21.15	36.82	73.51
LVDT3				
St-1LFRP-Off	351.0	8.3	25.44	3.1
St-1LFRP-On	345.0	7.8	22	2.8
Difference (%)	1.75	6.04	15.64	9.05
St-2LFRP-Off	358.7	10.1	29.2	2.9
St-2LFRP-On	364.9	9.2	14.43	1.6
Difference (%)	−1.71	9.20	102.36	85.32

**Table 4:** Comparison between codes and test results.

ACI (Force Unit: kN)					
Specimen	$V_{c}$	$V_{s}$	$V_{FRP}$	$P_{ACI}$	$P_{ACI}/P_{Exp}$
NoSt-NoFRP	70.8	0.0	0.0	106.3	0.54
NoSt-NoFRP	70.8	0.0	0.0	106.3	0.53
NoSt-1LFRP@90	70.8	0.0	40.7	167.3	0.76
NoSt-2LFRP@90	70.8	0.0	80.8	227.5	1.05
NoSt-1LFRP@170	70.8	0.0	21.6	138.6	0.54
NoSt-2LFRP@170	70.8	0.0	42.8	170.4	0.66
St-NoFRP	70.8	112.5	0.0	275.0	0.66
St-1LFRP-Off	70.8	112.5	21.6	307.4	0.71
St-2LFRP-Off	70.8	112.5	42.8	339.2	0.77
St-1LFRP-On	70.8	112.5	21.6	307.4	0.75
St-2LFRP-On	70.8	112.5	42.8	339.2	0.83
				Average	0.71
				COV	22%
*fib* (force unit: kN)					
Beam	$V_{c}$	$V_{s}$	$V_{FRP}$	$P_{fib}$	$P_{fib}/P_{Exp}$
NoSt-NoFRP	73.8	0.0	0.0	110.6	0.56
NoSt-NoFRP	73.8	0.0	0.0	110.6	0.55
NoSt-1LFRP@90	73.8	0.0	43.1	175.3	0.79
NoSt-2LFRP@90	73.8	0.0	58.5	198.4	0.91
NoSt-1LFRP@170	73.8	0.0	29.1	154.3	0.6
NoSt-2LFRP@170	73.8	0.0	44.2	177.0	0.69
St-NoFRP	73.8	101.3	0.0	262.6	0.63
St-1LFRP-Off	73.8	101.3	29.1	306.2	0.71
St-2LFRP-Off	73.8	101.3	44.2	328.9	0.75
St-1LFRP-On	73.8	101.3	29.1	306.2	0.75
St-2LFRP-On	73.8	101.3	44.2	328.9	0.80
				Average	0.70
				COV	16%

## Data Availability

Research data are available from the authors upon request.

## Abbreviations

The following abbreviations are used in this manuscript:

$E_{s}$	Modulus of Elasticity of Steel (GPa)
$E_{CFRP}$	CFRP Modulus of Elasticity (GPa)
*L*	Beam Length (m)
$V_{c}$	Concrete contribution to the shear capacity (kN)
$V_{s}$	Steel contribution to the shear capacity (kN)
$V_{FRP}$	FRP contribution to the shear capacity (kN)
*a*	Shear span (mm)
$b_{w}$	Beam web width (mm)
*d*	beam effective depth (mm)
$d_{FRP}$	Distance from the top of the beam to the lower edge of the FRP (mm)
$f_{cu}$	Concrete compressive strength (MPa)
$f_{y}$	Steel yield strength (MPa)
*h*	beam height (mm)
$S_{FRP}$	Center-to-center spacing between the applied FRP discrete strips (mm)
$t_{FRP}$	FRP thickness (mm)
$w_{FRP}$	Width of the FRP strips (mm)
$\rho_{FRP}$	Ratio of FRP reinforcement
$\epsilon_{CFRP}$	FRP ultimate elongation
$\rho_{v}$	Transverse reinforcement ratio
$\rho_{s}$	Longitudinal reinforcement ratio
U	U-Jacketing configuration of discrete FRP strips
EB-FRP	Externally Bonded Fiber-Reinforced Polymers
CFRP	Carbon Fiber-Reinforced Polymers
RC	Reinforced Concrete
CA	Coarse Aggregates
FA	Fine Aggregates
OPC	Ordinary Portland cement
LVDT	Linear Variable Displacement Transducer
$P_{y}$	Load capacity at yielding (kN)
$d_{y}$	Deflection at Yielding (mm)
$d_{m}$	Deflection at Failure (mm)
μ	Displacement ductility
COV	Coefficient of Variation
